# Psychopharmacological effects of riparin III from *Aniba riparia* (Nees) Mez. (Lauraceae) supported by metabolic approach and multivariate data analysis

**DOI:** 10.1186/s12906-020-02938-z

**Published:** 2020-05-16

**Authors:** Sócrates Golzio dos Santos, Isis Fernandes Gomes, Adriana Maria Fernandes de Oliveira Golzio, Augusto Lopes Souto, Marcus Tullius Scotti, Josean Fechine Tavares, Stanley Juan Chavez Gutierrez, Reinaldo Nóbrega de Almeida, José Maria Barbosa-Filho, Marcelo Sobral da Silva

**Affiliations:** 1 Instituto de Pesquisa de Fármacos e Medicamentos – IPeFarM, Universidade Federal da Paraíba, João Pessoa, PB 58051-900 Brazil; 2 Departamento de Tecnologia de Alimentos, Centro de Tecnologia e Desenvolvimento Regional, Universidade Federal da Paraíba, João Pessoa, PB 58051-900 Brazil

**Keywords:** Metabolomics analysis, Footprint, Urine, *Aniba riparia*, Riparin, Anxiety animal model

## Abstract

**Background:**

Currently there is a high prevalence of humor disorders such as anxiety and depression throughout the world, especially concerning advanced age patients. *Aniba riparia* (Nees) Mez. (Lauraceae), popular known as “louro”, can be found from the Amazon through Guianas until the Andes. Previous studies have already reported the isolation of alkamide-type alkaloids such as riparin III (O-methyl-N-2,6-dyhydroxy-benzoyl tyramine) which has demonstrated anxiolytic and antidepressant-like effects in high doses by intraperitoneal administration.

**Methods:**

Experimental protocol was conducted in order to analyze the anxiolytic-like effect of riparin III at lower doses by intravenous administration to Wistar rats (*Rattus norvegicus*) (*n* = 5). The experimental approach was designed to last 15 days, divided in 3 distinct periods of five days: control, anxiogenic and treatment periods. The anxiolytic-like effect was evaluated by experimental behavior tests such as open field and elevated plus-maze test, combined with urine metabolic footprint analysis. The urine was collected daily and analyzed by ^1^H NMR. Generated data were statistically treated by Principal Component Analysis in order to detect patterns among the distinct periods evaluated as well as biomarkers responsible for its distinction.

**Results:**

It was observed on treatment group that cortisol, biomarker related to physiological stress was reduced, indicating anxiolytic-like effect of riparin III, probably through activation of 5-HT_2A_ receptors, which was corroborated by behavioral tests.

**Conclusion:**

^1^H NMR urine metabolic footprint combined with multivariate data analysis have demonstrated to be an important diagnostic tool to prove the anxiolytic-like effect of riparin III in a more efficient and pragmatic way.

## Background

Currently there is a high prevalence of humor disorders, such as anxiety and depression [1, 2]; concerning advanced age patients, these psychiatric disorders are considered even more prevalent [3]. Many factors are involved in the etiology of anxiety, including genetics, gender, brain chemistry and incontrollable stressful events [4]. Previous research have demonstrated that medicinal plants have anxiolytic-like activity [5–10] and have been successfully used by folk medicine [11]. *Aniba riparia* (Nees) Mez. (Lauraceae) is a popular plant well known as “louro”, found from the Amazon and Guianas until the Andes, rich in Alkamide-type alkaloids, which were isolated from the green fruit of this species, such as riparin III (O-methyl-N-2,6-dyhydroxy-benzoyl tyramine) [12–14]. Previous in vivo studies have demonstrated anxiolytic and antidepressant-like effects of riparin III in high doses by intraperitoneal and oral administration [15]. Moreover, alkamides with similar chemical structures, such as riparin I and II have already demonstrated antinociceptive [16, 17] anxiolytic [18] and antidepressant-like effects [19]. Besides, semisynthetic chemicals (riparin A) have also proved its anxiolytic-like effects [20], supporting the important pharmacological activity of riparin and its variations.

Metabolomics is the science that studies the endogenous metabolites as a reflection of pathophysiological processes [21–24]. These metabolites play an important role as biomarkers, once its dynamic balance at biological fluids and tissues indicates a homeostasis state [25, 26]. In this context, abnormal metabolite processes or alterations in the metabolic balance caused by exogenous products characterize a certain profile that can be analyzed by techniques such as nuclear magnetic resonance (NMR) and mass spectrometry (MS) [21]. NMR is a technique that provides qualitative and quantitative measures of several compounds contained in a complex matrix [27]; unlike MS, NMR does not require complex pre-treatment of samples. Recently, numerous techniques have been developed in order to characterize metabolites and biological fluids [28]. The data obtained from these techniques treated with a chemometric approach can be used for clinical applications or as diagnostic tools [29], therefore, metabolomics platform associated with principal component analysis (PCA) [30] has demonstrated to be a very useful method in order to interpret multivariate data generated from the analyses of biological matrixes such as urine [31–33]. No bioanalytical method regarding determination of metabolic profile of anxious and stressed rats has been previously reported in the literature, as well as the detection of biomarkers correlated to riparin III administration at lower doses. Therefore, this present study has demonstrated the psychopharmacological effect of riparin III, through metabolic profiling of rats urine samples, performed by ^1^H NMR combined with multivariate data analysis.

## Methods

### Chemicals and reagents

Riparin III was obtained by organic synthesis and donated from Dr. Stanley Juan Chavez Gutierrez, and Dr. José Maria Barbosa Filho from Universidade Federal da Paraíba, Brazil, according to methodology previously described in literature [34].

During the whole experiment it was used ultra-pure water (Type I) obtained by Option-Q Purelab labwater-system (Elga, São Paulo, Brasil), deuterated water with sodium salt of 3-(trimethylsilil) propionic acid-d_4_ (TSP, 0.1% [w/v] in D_2_O) (Sigma-Aldrich, Brasil). Phosphate buffer (0.2 M Na_2_HPO_4_/NaH_2_PO_4_, pH 7.4).

### Standard solutions preparation

Riparin III was diluted in ultra-pure water with 0.1% of cremophor EL (Sigma-Aldrich, Brazil) in order to obtain an equivalent solution to a 5 mg.kg^− 1^ dose, which was injected in each rat the correspondent value in volume of the solution.

Riparin III was also diluted with 50 μL of acetonitrile, HPLC grade (Tedia, Brazil), and phosphate buffer 0.2 M in order to obtain a final solution of 1 mg.mL^− 1^ as NMR reference standard using the PRESAT method and TOCSY. Another sample of riparin III was also diluted with 50 μL of acetonitrile and rat urine in order to obtain a solution of 1 mg.mL^− 1^ as NMR reference standard which was also evaluated by the PRESAT method and TOCSY, this same procedure was repeated using water instead of urine for further observation of chemical shifts.

### Animals

The wistar rats (*Rattus norvegicus*), weighing approximately 250 g, 90 days old, were obtained from the Prof. Dr. Thomas George bioterium and maintained on standard laboratorial conditions: controlled temperature (21 ± 1 °C), 12 h light-dark cycle (lights on from 07:00 AM to 7:00 PM), with “pellet” type diet (Presence© – rats and mice, Brazil) and water ad libitum*.* The whole experiment was approved by the ethics committee in animal research (ECAR) under protocol n° 0107/08, from the Universidade Federal da Paraíba, João Pessoa/Paraíba, Brazil. The protocols are in agreement with the guidelines of the National Institute of Health (NIH Publication, Health Research Extension Act of 1985 Public Law 99–158, November 20, 1985 “Animals in Research”) regarding animal care in laboratory.

### Experimental protocol

**Fig. 1:**
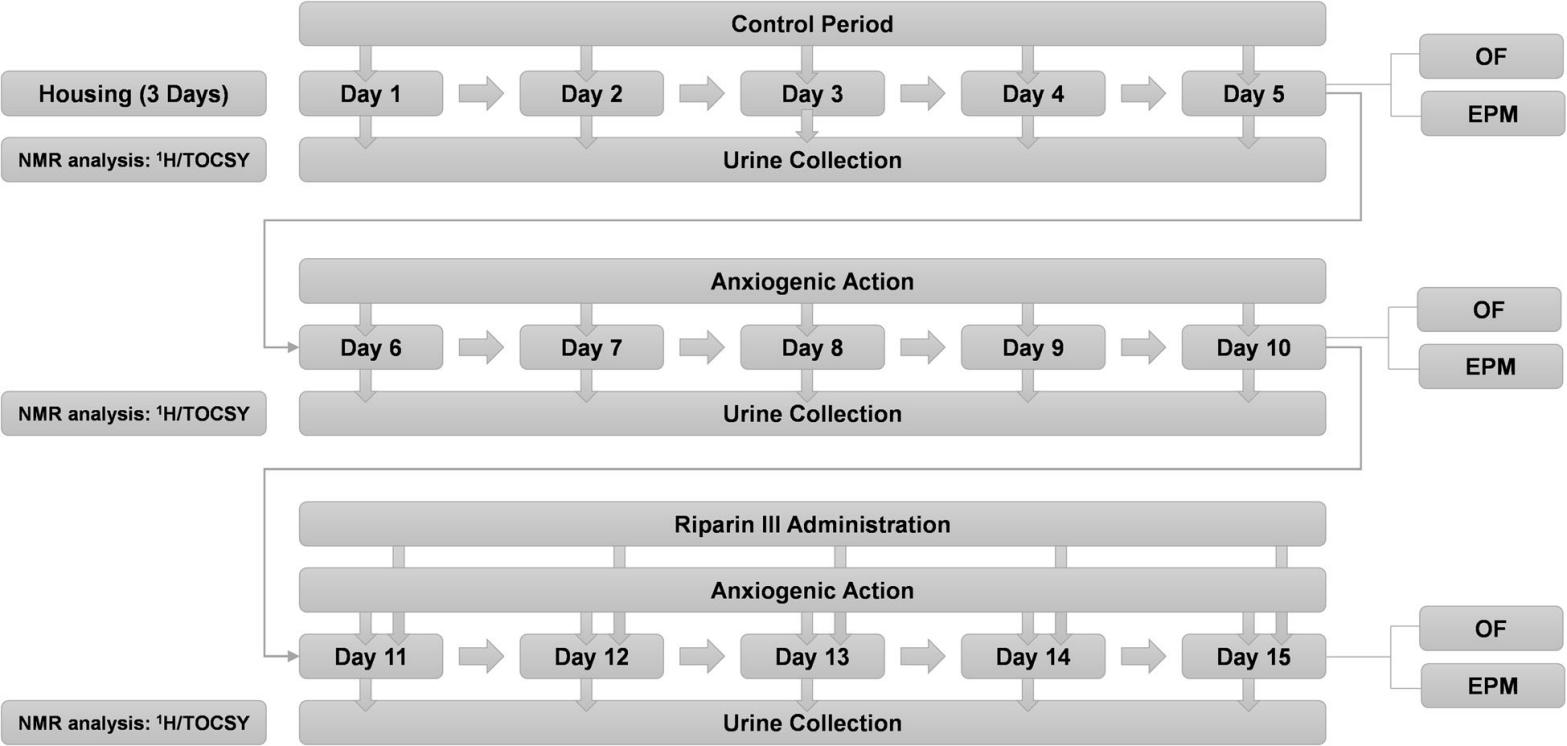
Experimental design concerning animal manipulation and sample collection

Firstly, the experimental protocol was performed with five Wistar rats, which were set in metabolic cages for 3 days [35], individually housed and maintained on standard laboratorial conditions. After 3 days, the animals remained for 5 days in its cages with the same conditions, with urine collection in every 24 h, for further analysis; this period was termed as control period (CP). The method was adapted from Aikey, Nyby [36], Broadhurst [37] and Broadhurst [38]. After the control period (CP), on the 5th day, the animals were submitted to the open field (OF) and elevated plus-maze tests (EPM), followed by the anxiogenic period (AP), which lasted 5 more days. The animals of AP went through food restriction in alternate days (24 h with food, followed by 24 h without food) [39, 40]. During this period, the animals were also placed collectively in the open field instrument and were submitted in a certain period of the day (10:30 a.m. - 03:00 GMT) to a sequence of sounds, from 5 Hz to 90 kHz, with an intensity of 80 dB, for 10 min [41, 42], these same procedure was repeated in the afternoon (4:30 p.m. − 03:00 GMT). The animals also went through forced swimming in plastic container (37 cm of diameter and 42. 5 cm of height, containing 30 cm of water a 25 ± 2 °C), individually, once a day, for 3 min. After each test, the animals were retrieved, dried and returned to its respective cages [43], the execution of the procedure was conducted twice a day, during the morning (10:30 a.m. − 03:00 GMT) and afternoon (4:30 p.m. − 03:00 GMT). On the 5th day of AP, the animals went through open field and elevated plus-maze tests. Posteriorly the animals were submitted to a period termed as treatment period (TP), for five more days, where they went through the same manipulation of AP, with an additional intravenous administration of riparin III, every day at 10:15 a.m. Finally, on the 5th day of TP, the animals were also submitted to open field and elevated plus-maze tests. After these experiments, the animals were anesthetized with ketamine hydrochloride (150 mg/kg), by intramuscular administration and xylazine hydrochloride (11 mg/kg) by intraperitoneal administration in order to eliminate the corneal reflex [44], after verification of the absence of this reflex, the animals suffered euthanasia by potassium chloride administration (2 mmol/Kg) followed by cervical dislocation [45]. Experimental design concerning animal manipulation and sample collection is demonstrated in Fig. 1.

### Behavioral tests

#### Elevated plus-maze test

The elevated plus-maze test (EPM) was conducted with a plus-shaped apparatus (10 × 10 cm), made of acrylic, with two open (50 × 10 × 0.5 cm) and two enclosed arms (50 × 10 × 42 cm), slightly elevated (54,5 cm), specifically designed to study behavioral changes predominantly associated with anxiety [46], once general behavior of rodents related to anxiety is the avoidance of open and elevated spaces [47, 48]. For behavioral evaluation, rodent was placed at the central platform facing the open arm, to manually measure the number of entries in the open and closed arms, as well as the time of permanence in both open arms, during the 5 min test [49]. The entry to one arm was only scored when the four paws of the animal were fully inside. During the 5 min test the behavior of rodent was recorded by video camera (SONY, model DSC-W180).

#### Open field test

The open field test (OF) was performed with a specific apparatus from Insight Equipamentos Científicos, model EP 154, made with acrylic, white floor with 70 cm^2^ area, delimited by black stripes in order to keep animal locomotion score. The field is constituted of two concentric circles, one circle with 30 cm of diameter and the other with 60 cm, in which the latter is delimited by a transparent cylinder of 50 cm (height) × 60 cm (diameter) with an illumination level of 225 1x. The behavior was scored according to definitions previously stablished [50]: the data were registered according to the number of ambulations (number of squares explored by each mouse with the four limbs), grooming, rearing and defecation. During the 5 min test, the animal behavior was recorded by video camera (SONY, model DSC-W180).

### Urine collection

This experiment was conducted for 15 days (excluding the first 3 days), all animals were weighed, the ration was measured during feeding days for consumption observation, volume of consumed water was also measured every 24 h, as well as the urinary volume and its pH, monitored with Merck tape. Aliquots of 300 μL of urine from each rodent were sampled for instant analysis every 24 h.

### Measure of urine samples by ^1^H NMR spectroscopy

To each aliquot (300 μL) of urine, from each animal, 300 μL of phosphate buffer were added (0,2 M Na_2_HPO_4_/NaH_2_PO_4_, pH 7.4) in order to minimize chemical shift variation. After mixing, the sample was homogenized in vortex for 30 s, followed by centrifugation at 14000 x *g*, during 10 min, at 15 °C. The supernatant (500 μL) was retrieved and mixed with 60 μL of D_2_O with TSP 0.1% [w/v], for “locking” field frequency and the sodium salt of 3-(trimethylsilil) propionic acid-d4 (TSP, 0.1% [w/v] em D_2_O) was used as internal standard, as chemical shift (δ 0) reference. The aliquots were transferred to 7 mm NMR tubes [51], and all the spectra were determined by a 500 mHz spectrometer from Varian, operating at 30 °C and spin frequency of 20 Hz. Unidimensional spectra were acquired by the PRESAT methodology with the following parameters: spectral window 8012.8 Hz, 32 K complex data points, 64 transients, acquisition time 2.3 s, relaxation time 2.0 s, pulse observation 4.56 μs with a 45° angle, line-broadening of 0.5 Hz. Additional conditions such as saturation time at 1.5 s and saturation power at 10 dB were stablished to suppress the water signal [52, 53]. Confirmation of the metabolites structure was achieved by bidimensional spectra acquired with TOCSY methodology following the parameters: spectral window 8012.8 Hz, 32 K complex data points, 8 transients, acquisition time 2.3 s, relaxation time 2.0 s, pulse observation 4.56 μs with a 45° angle, line-broadening of 0.5 Hz and additional conditions such as saturation time of 1.5 s and presaturation of the water peak with saturation power at 10 dB for suppression. Confirmation of the substances were also done by literature research.

### Statistical analysis

#### Statistical analysis of ^1^H NMR spectra from urine samples

Spectral intensities (peaks) were integrated into regions or binnings of equal width (0.02 ppm) comprising the range from 0.005 to 10 ppm. Spectral regions, corresponding to residual water (δ 4.75) were suppressed from all samples, in order to avoid efficiency variation of the process. Spectra were referenced to TSP as internal standard [53]. The ^1^H NMR spectroscopic data were reduced into 496 integral segments of equal length (0.02 ppm) and were exported into ASCII format to produce a data matrix of sample versus integral segments. Integrated areas were normalized to equal the total area and were submitted to Principal Component Analysis, using “The Unscrambler”, version 9.7 as statistical software (CAMO Process AS, Norway).

#### Statistical analyses of anxiety parameters from open field test, elevated plus-maze test, physiologic parameters and metabolic peaks

The data obtained were evaluated with one-way ANOVA followed by Bonferroni post hoc test, results with *p* values < 0.05 were considered statistically significant.

## Results

The elevated plus-maze is a validated test designed to evaluate the effects of anxiolytic drugs, once rodents have natural fear of heights and open spaces, therefore, in this particular test, the fact that the animal avoids open arms, characterize an anxiety-like behavior [47, 54].

**Fig. 2:**
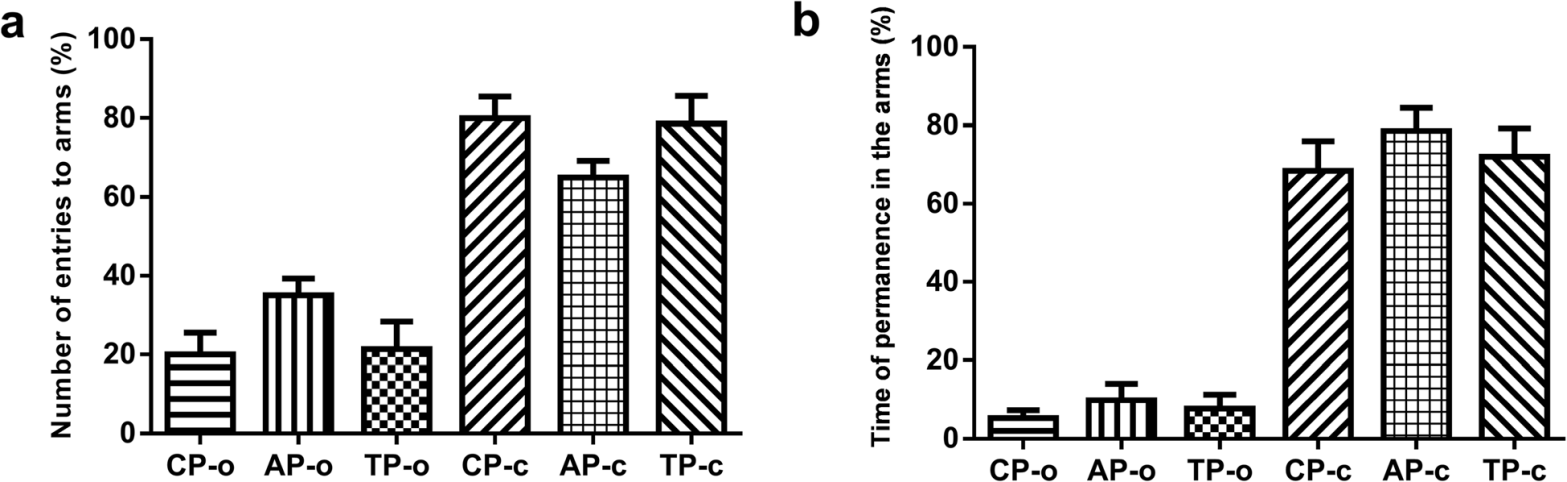
Elevated Plus-maze test. Numbers of entries to arms (**a**). Time of permanence on the open and closed arms (**b**). CP-o: Control Period (open arms). CP-c: Control Period (closed arms).AP-o: Anxiogenic Period (open arms). AP-c: Anxiogenic Period (closed arms). TP-o: Treatment Period (open arms). TP-c: Treatment Period (closed arms)

The results have demonstrated that there was no significant difference among CP, AP and TP groups, considering the number of entries on open arms. The same was observed concerning closed arms entries. However, the number of entries on closed arms by all tested groups were significantly higher than the number of entries on open arms by its respective groups (Fig. 2a).

Regarding the remaining time of the animals on open and closed arms, there was no significant change among groups. However, CP, AP and TP groups have remained more time on closed arms than its respective groups on open arms (Fig. 2b).

Therefore, the TP group, treated with Riparin III did not present a decrease regarding the number of entries or remaining time on closed arms when compared to CP and AP groups, indicating an anxiolytic-like effect.

Comparing altogether (number of entrances to open arms, closed arms and remaining periods), it could be observed that there was no alteration in associative memory and processing [55]. The use of drugs with hypnotic or sedative activity also diminishes mobility [56], in this context, treatments that block conditional fear may not necessarily block anxiety [57]. Therefore, stress would be defined as a threat to the homeostasis, which may be restored by a complex repertoire of physiological and behavioral responses concerning its adaptation [58].

The open field test is used to evaluate the locomotor activity of the animal as an essential parameter to analyze the drug effect towards central nervous system (CNS). On this specific test, the animal is submitted to a new environment, promoting a tendency to be explored, evoking fear and curiosity, being considered a behavioral model [59]. The principle of this test is based on the increasing number of ambulations and rearings frequencies, characterizing an anxiolytic-like behavior, while the reduction of locomotion stands for sedative effect [60].

**Fig. 3:**
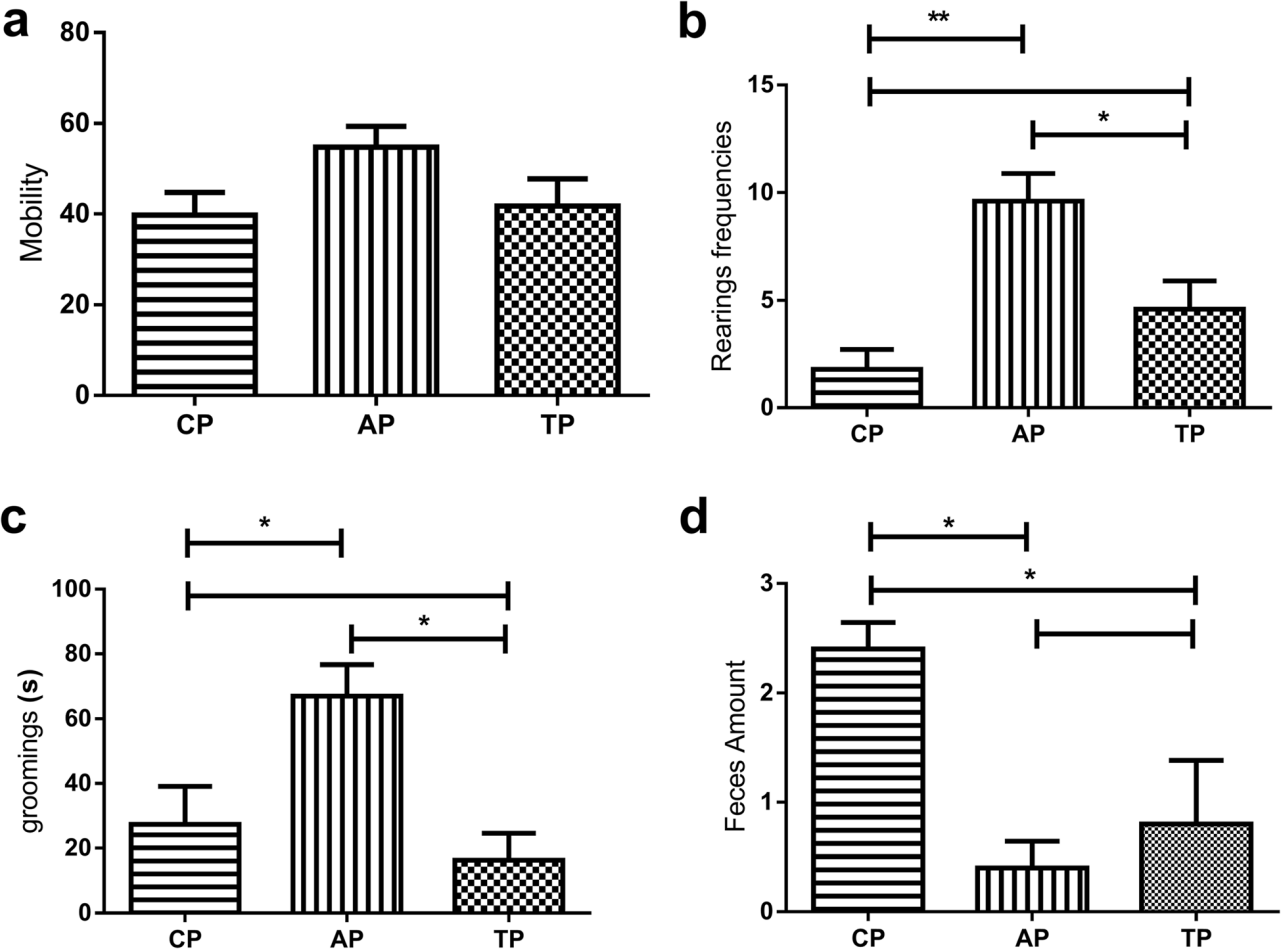
Open Field test. Mobility (**a)**. rearings (**b**). groomings (**c**). feces amount (**d**). CP: Control Period. AP: Anxiogenic Period. TP: Treatment Period

There was also observed no alteration of fear, when mobility was evaluated, showing no significant differences among periods (CP, AP and TP) (Fig. 3a), demonstrating that at this dose the drug has no hypnotic or sedative effect, as well as fear inhibition [61]. On Fig. 3b, it was observed that the treatment with riparin III (TP) has diminished significantly the rearing frequencies of the animals, when compared to AP, and has not presented significant difference when correlated with CP, suggesting that the drug at this dose caused alteration on alert behavior [62]. This same observation was corroborated by evaluation of grooming (Fig. 3c), which has decreased significantly when comparing TP to AP. Moreover, TP did not present significant alteration when related to CP, demonstrating anxiolytic-like effect of the drug [63]. Number of defecations (Fig. 3d) has decreased significantly when comparing CP to AP and TP, besides, no significant difference was observed between AP and TP, demonstrating that the anxiogenic facts have altered the animals emotionally [64, 65], leading only to activity decrease [66].

Concerning behavioral evaluation, it was observed that the anxiogenic factors have stressed the animals during the whole experiment, preserving the vigilant state and leading the animals to escape and seek for safe places [63], typical defensive reactions related to anxiety [67].

**Fig. 4:**
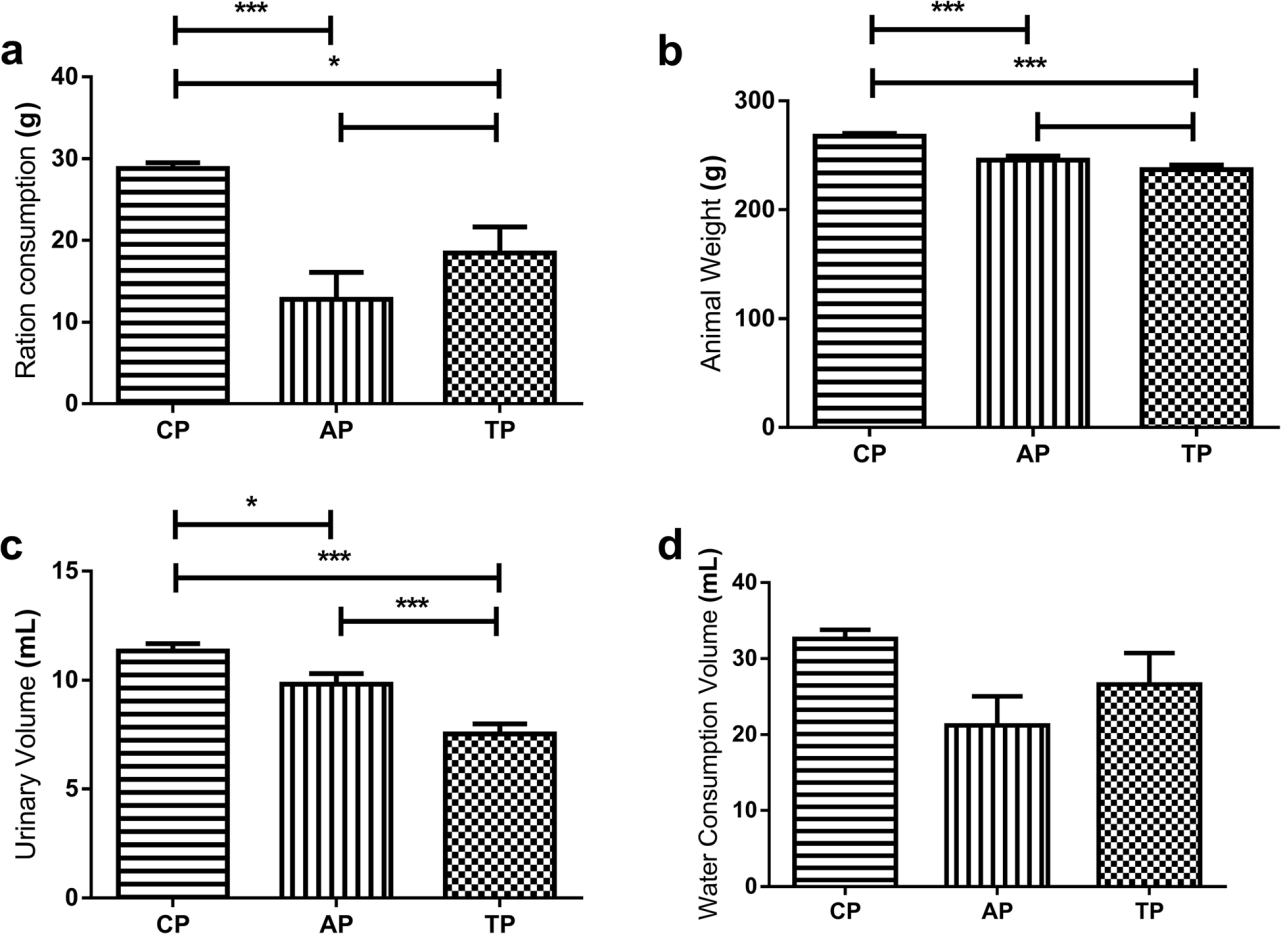
Evaluation of physiological parameters. Ration weight (**a**). Animal Weight (**b**). Urinary volume (**c**). Water consumption volume (**d**). CP: Control Period. AP: Anxiogenic Period. TP: Treatment Period

On Fig. 4a, concerning feeding, as physiological parameter, measured by ration consumption, there was observed significant difference between CP and AP (*p* < 0.0001), as well as, between CP and TP (*p* < 0.05), but no significant difference between AP and TP.

The difference related to CP is due to the null values, registered during feeding restriction time of 24 h, nevertheless, it was not observed the riparin III effect regarding hanger/feeding parameter [68], when comparing AP to TP.

On Fig. 4b, regarding weight, as physiological parameter, there was observed a significant difference between CP and AP (*p* < 0,0001), as well as, between CP and TP (*p* < 0.0001), but no significant difference between AP and TP; these differences presented, do not reflect directly on the anxiogenic factors or the riparin III towards the CP due to fee restriction of 24 h, suggesting the influence of innutrition towards substantial weight loss [69–72].

On Fig. 4c, regarding urinary volume, it was detected a significant difference between CP and AP (*p* < 0.05), CP and TP (*p* < 0.0001), as well as between AP and TP (*p* < 0.0001). These data have demonstrated that during anxiogenic period, physiological alterations decreased the average urinary volume eliminated by the animals, corroborating with the data of Ullrich, Lutgendorf [73]. The use of riparin III (5 mg.kg^− 1^) has reduced the average urinary volume [74]. Regarding bladder functions, considering that the micturition reflex is controlled by inhibitory mechanisms, and that the storage urine function is more important than micturition reflex by evolutionary reasons [75], the serotoninergic system may be involved at blocking mechanisms over afferent nerve of micturition reflex and urethra contraction, probably through glycinergic neurone inhibition, from lumbosacral marrow. Furthermore, the 5-HT_2A_ receptors may be involved on bladder and urethra functions as well. The results suggest that riparin III has effect over the 5-HT_2A_ receptors from amygdala [76], once this drug has demonstrated its anxiolytic-like effect on open field tests, by decreasing activity parameters, such as grooming and rearing (Fig. 3 B e D) [77]; however, it has presented no effect over reward system which has the 5-HT_1B_, 5-HT_2A_ and 5-HT_2C_ receptors [78].

Previous research has demonstrated that behavioral responses are related to visceral responses, which is reflected by the reduction of urinary volume after riparin III treatment [75, 79] proving stress treatment reduces urinary incontinence.

On Fig. 4d, physiologic parameters such as water consumption (24 h), did not demonstrate significant difference among periods (CP, AP and TP).

**Fig. 5:**
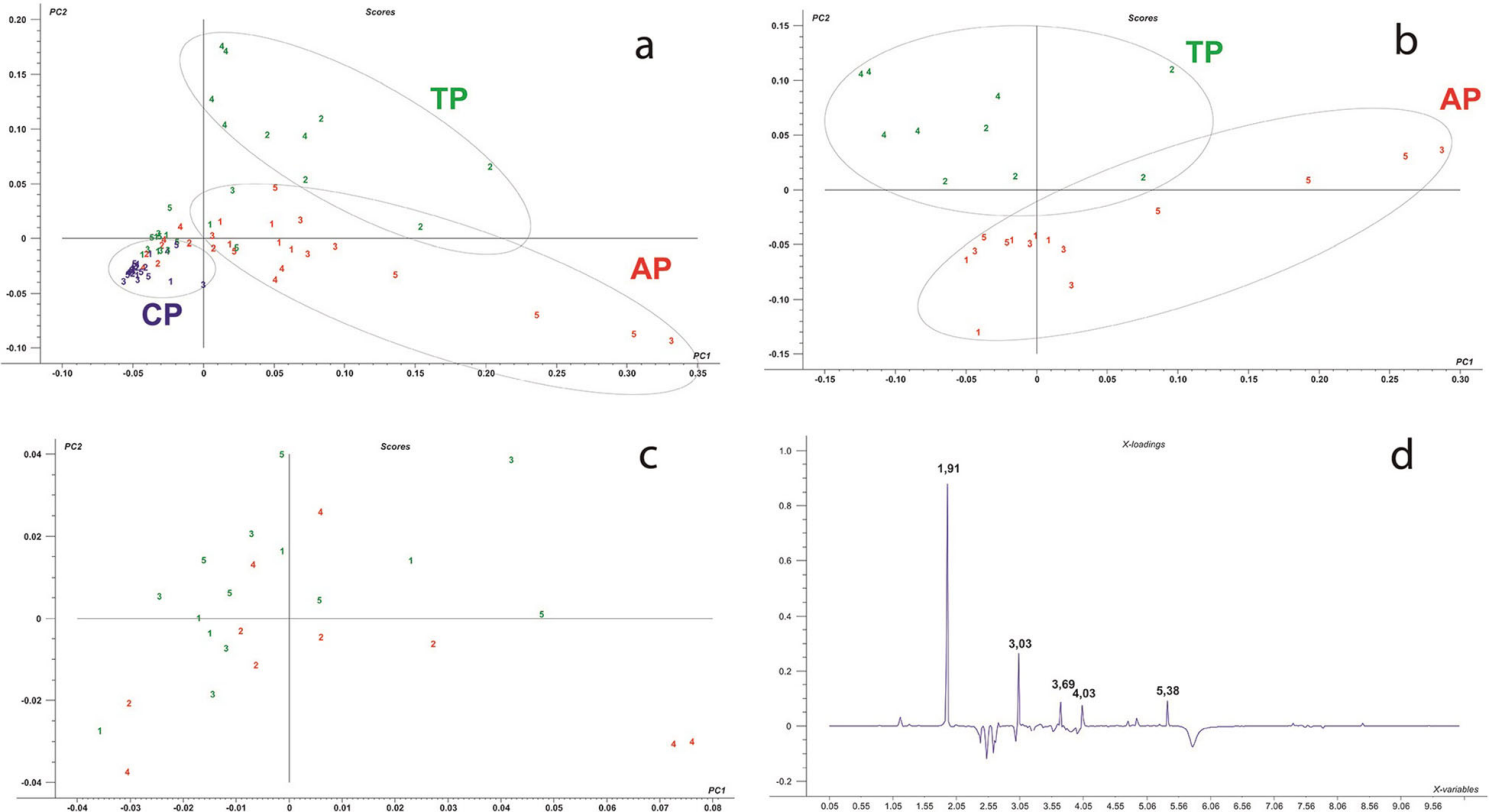
PCA Scores plot of urinary 1H NMR spectra regarding metabolite concentration (a), (b) and (c). The colors reveal the different analysis periods (CP – blue, AP – red, TP – green). Numbers represent the days of each analysis. (CP – Control period, AP – Anxiogenic Period and TP – treatment period). **a** Profiles of 3 analyzed periods. The best adjustment and predictability for these models were evaluated by R2 = 0.71 and Q2 = 0.89). **b** Profiles of AP and TP on days of food restriction. The best adjustment and predictability for these models were evaluated by R2 = 0.76 and Q2 = 0.85). **c** Profiles of AP and TP on days of ad libitum feeding. The best adjustment and predictability for these models were evaluated by R2 = 0.62 and Q2 = 0.83). **d** Loadings plot of urinary metabolites responsible for the differentiation of the 3 analyzed periods (CP, AP and TP)

Spectral analyses of ^1^H NMR from urine samples of CP, AP and TP (Fig. 5) were submitted to multivariate data analysis, using PCA as statistical tool in order to visualize and detect slight differences among the distinct periods [80–82].

PCA analysis was used in order to verify differences among samples and classify them according to its ^1^H NMR results, resulting in groups or clusters, reflecting on its distinct periods. According to the scores plot (Fig. 5a) it could be observed the formation of three distinct groups, classified as CP, AP and TP, which according to the loadings plot, (Fig. 5d) were differentiated by influence of five significant components [83]: cortisol (δ 1.91 s), creatinine (δ 3.03 s), riparin III (δ 3.69 s), 5-hydroxy-L-triptophan (δ 4.03dd) and allantoin (δ 5.38 s), which were identified with the aid of TOCSY technique and confirmed by scientific literature [84–87].

The statistical analysis on Fig. 5b has also demonstrated that during food restriction, when analyzing only AP and TP, the discrimination between groups was more evident, with a higher variance of AP along the PC1 dimension, and a R2 = 0.76. On the other hand, during ad libitum feeding the R2 correlation has diminished to 0.62 (Fig. 5c), indicating that feeding reduces stress.

**Fig. 6:**
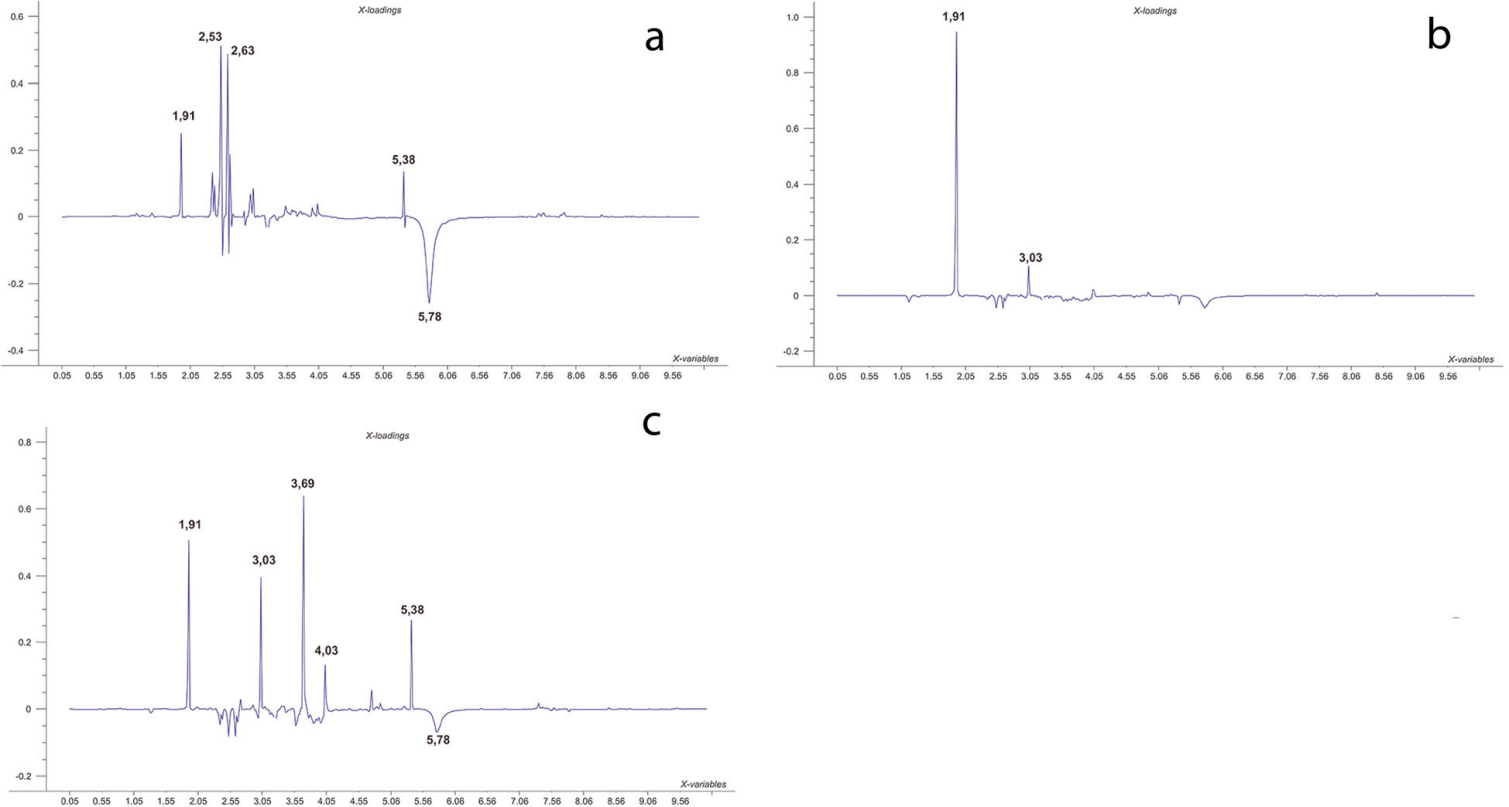
Main loadings variables responsible for group differentiation. Loadings variables of control period (CP) (**a**). Loadings variables of anxiogenic period (AP) (**b**). Loadings variables of Treatment Period (c)

It could be observed in the loadings plot (Fig. 6), variables or peaks responsible for the differentiation of each period or group. Regarding the loadings plot of control period (CP), on Fig. 6a, it was demonstrated that the most important peaks responsible for its segregation were δ 2.53 and δ 2.63 for cyanocobalamin (vitamin B12), δ 1.91 for cortisol, δ 5.38 for allantoin and δ 5.78 for urea, whereas the most influential peaks of the loadings plot concerning the anxiogenic period (AP) (Fig. 6b), were δ 1.91 for cortisol and δ 3.03 for creatinine. Moreover, in relation to the loadings plot of treatment period (Fig. 6c), it has demonstrated that the most influential peaks towards this particular group were δ 3.69 for riparin III, δ 1.91 for cortisol, δ 3.03 for creatinine, δ 5.38 for allantoin, δ 4.03 for tryptophan and δ 5.78 for urea.

The metabolic profile of control period (Fig. 6a) is basically defined by metabolites from food constituents, among which, the peaks δ 2.53 and δ 2.63 from cyanocobalamin were one of the most important components from the ration provided, rich in B-complex vitamins. The presence of the cortisol peak is suggestive due to the permanence of the animals in a cage, with no physical activity during the whole experiment [1]; regarding the peaks of allantoin (δ 5.38) and urea (δ 5.78), it would be due to the expected physiology of rodents, catabolizing nitrogenized products. The anxiogenic period (Fig. 6b) was characterized by the presence of cortisol (δ 1.91) and creatinine (δ 3.03), which might be related to physical effort, caused by forced swimming, besides food restriction [88–90], indicating that these factors would be related to significant weight loss presented on Fig. 4b, once bioterium animals are sedentary, therefore the presence of these metabolites were not significantly important on EPM (Fig. 2) or OF tests (Fig. 3a). Regarding TP profile, the cortisol peak (δ 1.91) still had influence over this period, demonstrating that anxiogenic factors have remained, corroborating with Fig. 2 and Fig. 3a. The presence of creatinine (δ 3.03) and allantoin (δ 5.38) have indicated high catabolism of proteins, due to food restriction, however, the presence of the peak related to riparin III (δ 3.69) was a very important parameter for this group, reflecting on the animal behavior of TP demonstrated by Fig. 3b, with the strong reduction of rearing and grooming (Fig. 3d) when compared to AP. Furthermore, the observation of no significant difference between TP and CP regarding these two specific behaviors has reinforced its anxiolytic-like effect [62, 66, 91].

The significant influence of tryptophan peak (δ 4.03) on TP (Fig. 6c) has suggested a deviation on metabolic path towards biosynthesis and/or storage of serotonin, once tryptophan is one of serotonin’s precursor [92]. ANOVA one-way analysis, applying Bonferroni post-test upon the tryptophan areas of urine samples (Fig. 8b) has demonstrated significant difference between CP and AP periods, as well as between CP and TP, but did not present significant difference between AP and TP. The peak δ 4.03 was predominantly detected on animals from the TP group, indicating that those animals were under anxiolytic-like effect [57]. However it was also detected on treated animals, a peak related to riparin III (δ 3.69) and a decrease related to cortisol (δ 1.91), indicating an antagonistic effect, or a reduction of cortisol levels due to riparin treatment, consequently inhibiting the anxiogenic induction.

**Fig. 7:**
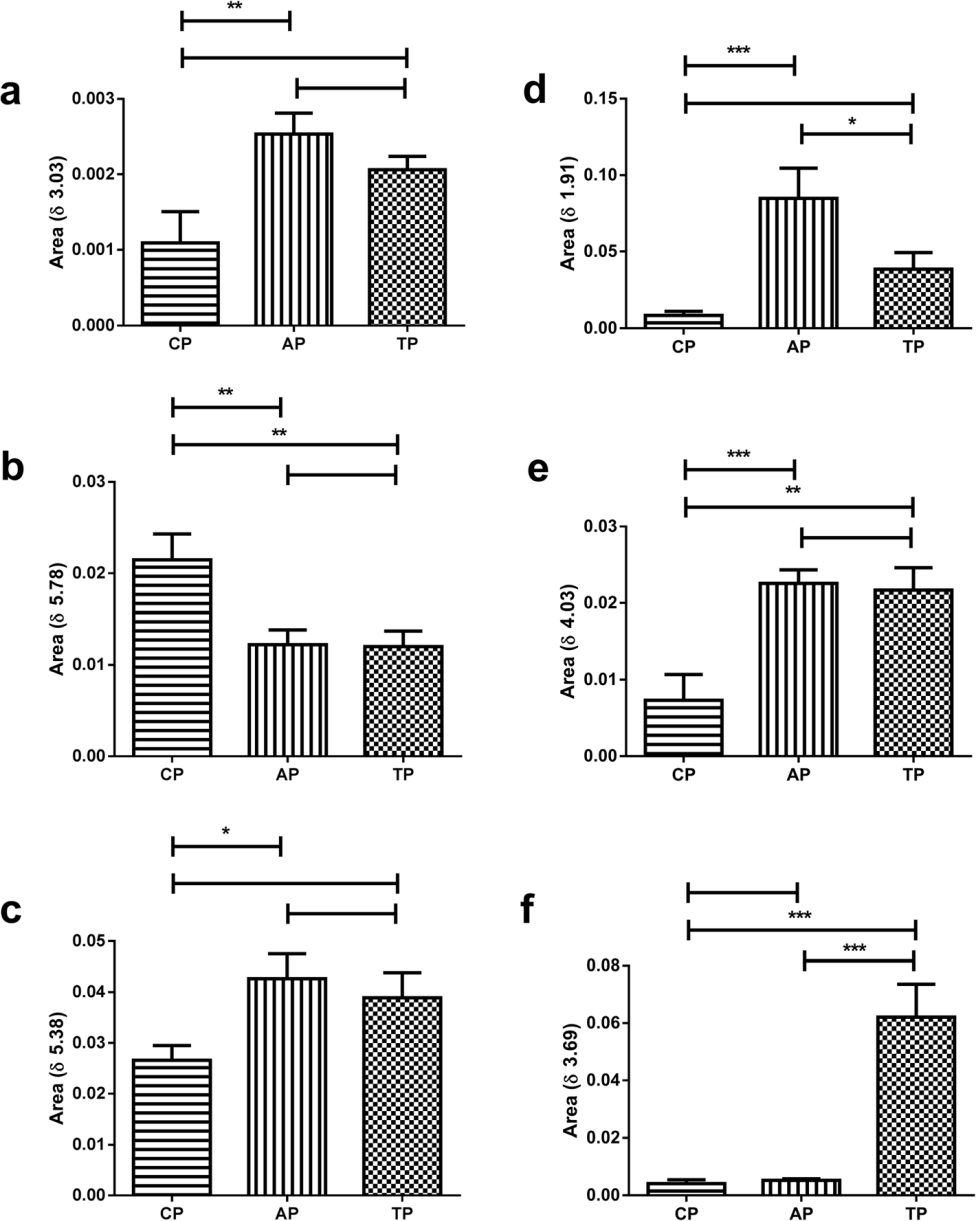
Creatinine (**a)**, Urea (**b**), Allantoin (**c**), Cortisol (**d**), Tryptophan (**e**) and Riparin peak (**f**) after physical effort and food restriction. CP: Control Period. AP: Anxiogenic Period. TP: Treatment Period. *p* < 0.05 (*). *p* < 0.001 (**). *p* < 0.0001 (***)

Regarding the urea peak (δ 5.78) on Fig. 6, it has presented a significant decrease on CP, when correlated to AP and TP, but it was not observed a significant difference between AP and TP, which would be due to physiological needs of the animals in order to obtain energy [93], once they went through physical effort and food restriction, corroborating with creatinine peaks at δ 3.03, observed on Fig. 7a, which has increased significantly on AP when compared to CP. The energetic needs were supplied by catabolism provided from muscular tissue [94]. The lack of significant difference between CP and TP, as well as between AP and TP (Fig. 7a), have suggested that during TP, there was a decrease concerning muscular stress, but no difference was detected on the previous period (AP) [95].

The Fig. 7 d and f have indicated an antagonistic effect, correlating cortisol (δ 1.91) and riparin III (δ 3.69), which were also supported by behavioral studies (Fig. 3 b and d). According to statistical analysis, it was observed a significant increase of riparin III (Fig. 7f) when comparing CP to TP, and AP to TP, besides, it was also observed a significant reduction of cortisol (Fig. 7d) when correlating AP to TP and no significant difference between CP and TP, demonstrating that the increase of riparin III has reduced the cortisol levels; consequently, the behavioral effects caused by cortisol (Fig. 3b and d) have diminished significantly due to the presence of riparin III, promoting anxiolytic-like effect [96, 97].

**Fig. 8:**
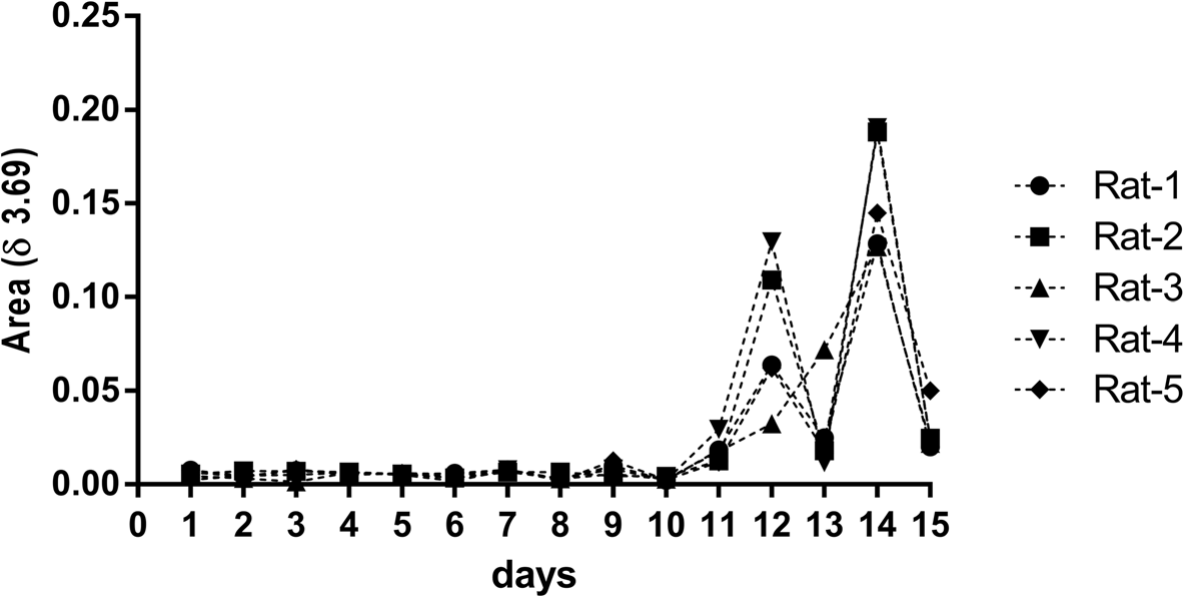
Riparin III peaks on urine samples between days 11 and 15. The x-axis represents the days of the analyses. Days 0 to 4: Control Period (CP), days 5 to 10: Anxiogenic Period (AP) and days 11 to 15: Treatment Period (TP)

On Fig. 8 it could be highlighted the increasing area of peak δ 3.69 from day 11 to 15, corresponding to TP, however, on days 13 and 15, it was observed smaller areas, closer to day 11. The animals went through food restriction on alternate days, from day 5 to 15, with no ration at odd days. Riparin III is strongly bonded to albumin, and pre-albumin, considered by previous studies an indicative parameter of chronic innutrition [98], and acute innutrition respectively [99], along with high levels of creatinine and allantoin (Fig. 7 a and c) on AP and TP periods. Therefore, suggesting that on odd days, during AP and TP periods, the animals suffered of acute innutrition aggravated by forced swimming. It was also observed that on days 12 and 14, peak area at δ 3.69 has increased to an average value of 0.0793 ± 0.0175 (Mean ± SEM) and 0.1558 ± 0.0141 (Mean ± SEM) respectively. Pre-albumin has a half-life of approximately 48 h [100, 101] the average value of the peak area at δ 3.69 has augmented 1.96 times when comparing day 12 and 14, however, on days 13 (0.0289 ± 0.0109) and 15 (0.0282 ± 0.0055), these values came closer to day 11 (0.0180 ± 0,0031). If on odd days, the concentration of pre-albumin drops due to the increase of catabolism caused by food restriction and physical effort, considering that its half-life is of 48 h and that on even days the anabolism increases due to food availability, consequently riparin III (δ 3.69) has a half-life up to 12 h, and the minor values at days 13 and 15 would be also due to the bond of riparin III with pre-albumin.

Previous research have demonstrated anxiolytic and antidepressant-like effect of riparin III in high doses by intraperitoneal administration [102]. In this work, the anxiolytic-like effect has been demonstrated at a lower dose by intravenous administration supported by metabolic profiling and behavioral tests. Moreover, it has been speculated that, due to its anxiolytic-like effect, riparin III in a 5 mg.kg^− 1^ dose acts on serotoninergic neurons [96] promoting a behavior controlled by amygdala [103], once previous studies have already indicated the role of amygdala on anxiety, specially its central core [57]. ^1^H NMR data of urine samples were statistically treated by principal component analysis in order to detect patterns among the distinct periods evaluated, as well as biomarkers responsible for its distinction [104]. Cortisol, biomarker related to physiological stress, poorly indicates the psychological state [105], however it was supported by not only behavioral tests such as OF and EPM, but physiologic parameters analyses, that the reduction of cortisol by riparin III was related to anxiety reduction probably through activation of 5-HT_2A_ receptors [75]. Previous study has demonstrated that behavioral responses are related to visceral responses [75], in this context, the reduction of urinary volume after riparin III treatment [79] was also observed, corroborating with the cortisol level reduction, proving that stress treatment reduces urinary incontinence.

## Conclusion

Anxiolytic-like effect of riparin III has been demonstrated by behavioral tests such as open field and elevated plus-maze tests. The results were obtained at a lower dose by intravenous administration to Wistar rats. Moreover, it was not observed any hypnotic or sedative effect towards the animals whatsoever, ergo, preserving the vigilant state. These results were also supported by metabolic profiling from urine samples, obtained by the combination of ^1^H NMR analysis and statistical treatment of its data by principal component analysis, which could detect cortisol, creatinine, allantoin and tryptophan as biomarkers. Therefore, urinary metabolic profiling by ^1^H NMR spectroscopy combined with multivariate data analysis have demonstrated to be an important diagnostic tool to prove the anxiolytic-like effect of riparin III in a more efficient and pragmatic way.

## Data Availability

The dataset generated during the present study is available upon reasonable request to the author (Prof. Marcelo Sobral).

## Abbreviations

OF: Open field
EPM: Elevated plus-maze
CP: Control period
AP: Anxiogenic period
TP: Treatment period
TSP: 3-Trimethylsilil propionic acid
PCA: Principal component analysis
